# The next-generation DNA vaccine platforms and delivery systems: advances, challenges and prospects

**DOI:** 10.3389/fimmu.2024.1332939

**Published:** 2024-02-01

**Authors:** Bowen Lu, Jing Ming Lim, Boyue Yu, Siyuan Song, Praveen Neeli, Navid Sobhani, Pavithra K, Srinivasa Reddy Bonam, Rajendra Kurapati, Junnian Zheng, Dafei Chai

**Affiliations:** ^1^ Cancer Institute, Xuzhou Medical University, Xuzhou, Jiangsu, China; ^2^ Jiangsu Center for the Collaboration and Innovation of Cancer Biotherapy, Cancer Institute, Xuzhou Medical University, Xuzhou, Jiangsu, China; ^3^ Center of Clinical Oncology, Affiliated Hospital of Xuzhou Medical University, Xuzhou, Jiangsu, China; ^4^ Department of Medicine, Baylor College of Medicine, Houston, TX, United States; ^5^ Department of Environmental Science, Policy, and Management, University of California at Berkeley, Berkeley, CA, United States; ^6^ Department of Neuroscience, Baylor College of Medicine, Houston, TX, United States; ^7^ School of Chemistry, Indian Institute of Science Education and Research Thiruvananthapuram, Thiruvananthapuram, India; ^8^ Department of Microbiology and Immunology, University of Texas Medical Branch, Galveston, TX, United States

**Keywords:** DNA vaccines, delivery system, viral vectors, non-viral vectors, nanoparticles

## Abstract

Vaccines have proven effective in the treatment and prevention of numerous diseases. However, traditional attenuated and inactivated vaccines suffer from certain drawbacks such as complex preparation, limited efficacy, potential risks and others. These limitations restrict their widespread use, especially in the face of an increasingly diverse range of diseases. With the ongoing advancements in genetic engineering vaccines, DNA vaccines have emerged as a highly promising approach in the treatment of both genetic diseases and acquired diseases. While several DNA vaccines have demonstrated substantial success in animal models of diseases, certain challenges need to be addressed before application in human subjects. The primary obstacle lies in the absence of an optimal delivery system, which significantly hampers the immunogenicity of DNA vaccines. We conduct a comprehensive analysis of the current status and limitations of DNA vaccines by focusing on both viral and non-viral DNA delivery systems, as they play crucial roles in the exploration of novel DNA vaccines. We provide an evaluation of their strengths and weaknesses based on our critical assessment. Additionally, the review summarizes the most recent advancements and breakthroughs in pre-clinical and clinical studies, highlighting the need for further clinical trials in this rapidly evolving field.

## 1 Introduction

Vaccines have made significant progress since the 20th century. Early successes include the smallpox vaccine, inactivated polio vaccine, and the first recombinant subunit hepatitis B vaccine (1, 2). Historical vaccines can be categorized into more than three types: live attenuated, inactivated, subunit/recombinant subunit, and others (3–5). The development of microbiology in the mid-20th century facilitates the rapid advancement of subunit vaccines, leading to the creation of vaccines for diseases such as mycobacterium leprosy and malaria (6, 7). While vaccines offer substantial benefits, they also entail certain risks. Live attenuated vaccines, such as measles, mumps, and rubella (MMR), varicella, rotavirus, BCG, and others, have undergone selective mutation to lose their pathogenicity (8). However, there is still a potential for them to retain pathogenicity and cause adverse immune responses (9). Inactivated vaccines, although not capable of restoring virulence, have complex preparation processes, risk of contamination, and potential issues with incomplete inactivation. Concerns about pollution, potency stability, local and systemic reactions, as well as adverse effects, must be carefully considered when selecting vaccines for further development (10, 11). Subunit vaccines and recombinant subunit vaccines, on the other hand, are considered safe due to the absence of external additives and the use of recombinant technology instead. However, they face challenges regarding low immunogenicity, which can be addressed by adding adjuvants (12). While traditional vaccine therapies have shown effectiveness in inducing immune responses and yielding therapeutic benefits, they encounter various challenges when dealing with certain diseases.

In 1990s, a preclinical study determined that intramuscular plasmid DNA injection could stimulate protein expression, and it was demonstrated that the majority of cells could transport DNA to the nucleus without the need for a specialized delivery system (13). Since their introduction in the 1990s, DNA vaccines have proven to be scalable, stable, and flexible. The initial iterations of gene vaccines comprised solely DNA in the form of plasmids, which are cellularly internalized and transcribed into protein (14). DNA vaccines, on the other hand, are genetically engineered DNAs that serve as templates to transmit molecular information and trigger antigen-specific immune responses. These vaccines not only encode the target antigen but also rely on an effective delivery system to introduce the target antigen gene and express the corresponding protein for immune response activation. For example, neoantigens are a class of antigens that arise from somatic mutations in tumor cells. These mutations create novel peptide sequences that are not present in normal cells, making them unique targets for the immune system to recognize and attack cancer cells (15). Therefore, there has been a growing focus on neoantigen-based DNA vaccines in the field of immunization. The selection of effective neoantigens and their efficient delivery to the immune system is crucial in achieving accurate and protective immunity with minimal side effects (16). In this review, we discuss the advancements in vector-engineering efforts, immune stimulants, delivery routes, and injection methods that contribute to the development of next-generation DNA vaccines. Furthermore, we summarize and analyze ongoing clinical trials involving DNA vaccines that utilize various delivery systems.

## 2 Current status and challenges of DNA vaccine

Nowadays, vaccine development has progressed to three generations. The first generation consists of weakened or inactivated vaccines, the second generation involves protein subunit vaccines that present a reduced risk of infection, and the third generation encompasses DNA and RNA, virus-like particle (VLP), protein- and plant-based vaccines that produce the antigen within the body (17). In comparison to protein-based vaccines, which typically present antigens through phagocytosis, and cellular vaccines, which immediately present antigens after vaccination, DNA-based vaccines undergo transcription and/or translation processes to present antigens via both major histocompatibility complex (MHC) I and II pathways (18). Compared to traditional vaccines, DNA vaccines offer several advantages. Firstly, they have relatively low manufacturing cost, easy production processes, high stability, and a good safety profile (19). In contrast to conventional protein antigens, which require complex purification methods, different DNA constructs have stable physical and chemical properties and can be purified through a single procedure. This makes them more convenient to store, transport, and distribute, even in remote areas. Additionally, DNA vaccines are not affected by the potential virulence transformation associated with live attenuated vaccines or the side effects commonly associated with inactivated cell vaccines. Secondly, DNA vaccines have flexible construction and can be easily manipulated using molecular technology. The designed DNA vaccine can specifically express the antigen of interest or integrate genes encoding multiple antigens into a plasmid, providing immune protection against multiple diseases. For instance, DNA vaccine can be introduced with chimeric cytokine genes as adjuvants into the antigen plasmid, allowing for co-expression and enhanced immune responses (20, 21). Furthermore, specific motifs on the DNA construct can induce immunostimulatory effects, even in the absence of antigen-coding genes (22). Lastly, DNA vaccines can elicit a broad spectrum of the immune responses, including both humoral immunity (involving antibodies) and cellular immunity (involving T cells). The induction of cellular immunity is particularly important for diseases that require a robust cellular immune response, such as anti-tumor immunity (23). Moreover, DNA vaccines have shown effectiveness in inducing protective immunity against viral infections (24).

Although DNA-based treatments offer many advantages, concerns have been raised in the past regarding the potential risk of integrating the host genome (25). There is a theoretical possibility that this DNA could integrate into the host genome, potentially leading to unintended genetic changes. However, it is important to note that DNA vaccines are designed to minimize the risk of genetic integration (26). The DNA used in vaccines is typically in the form of a circular plasmid (27), which is different from the linear chromosomes found in the host genome. DNA has a lower tendency to integrate into the host genome compared to linear DNA. Furthermore, the amount of DNA delivered by a DNA vaccine is usually very small (28), reducing the likelihood of integration. Additionally, the delivery method used for DNA vaccines, such as injection or electroporation (29), targets specific cell types, such as muscle cells or dendritic cells (DCs) (28), minimizing the exposure of other cell types to the vaccine. Numerous preclinical and clinical studies have been conducted to evaluate the potential for genetic integration of DNA vaccines, and the overall evidence suggests that the risk of integration is low (30, 31). However, it is important to continue monitoring and conducting research on DNA vaccines to ensure their safety. Regulatory authorities have specific guidelines and requirements in place to assess the safety and potential for genetic integration of DNA vaccines during their development and approval processes. It is important to note that when DNA vaccines are applied to large mammals or humans, they may face limitations. However, these limitations can be addressed through the use of cytokine-coding nucleic acids, codon optimization or delivery systems, which can help overcome the insufficiencies (32, 33). Adjuvants act as additional immune stimulator that promote the maturation and activation of immune cells upon antigen introduction. Traditional adjuvants like alum enhance antigen presentation by adsorbing antigens on their surface, activate the NLRP3 inflammasome, and modulate immune responses through cytotoxic actions. Co-administration of DNA vaccine with other adjuvants, such as cytokines, bacterial proteins, or fusion of the gene sequence encoding the adjuvant into the plasmid DNA, can effectively improve immunogenicity and elicit a strong immune response (34–36). Furthermore, synthetic oligonucleotides at the nucleic acid level have shown promise as vaccine adjuvants. Among these, CpG DNA, which mimics the structure of bacterial DNA, acts as a toll-like receptor (TLR) agonists and activate antigen-presenting cells (APCs) through the innate immune response. This activation enhances the immune response to the vaccine (37). Additionally, modifications to nanocarriers have emerged as a growing field in vaccine development (38). These modifications allow nanocarriers to serve as self-adjuvant nanomaterials rather than just acting as carriers. They can activate APCs, leading to the production of cytokines and chemokines that further promote immune responses. Self-adjuvant nanomaterials not only improve the stability and delivery of DNA vaccines but also enhance the overall immune response. More importantly, they can be engineered to target specific immune cells or tissues, increasing their effectiveness and specificity. These advancements in adjuvant technologies and nanocarrier modifications contribute to the development of more effective DNA vaccines by improving immune responses, reducing the required vaccine dosage, and allowing for targeted delivery to specific immune cells or tissues. Continued research in these areas holds great potential for further enhancing the efficacy and safety of DNA vaccines.

In recent years, there has been increasing interest in the development of self-adjuvant nanomaterials for DNA vaccines (39). These nanomaterials possess intrinsic adjuvant properties, eliminating the need for additional adjuvant components. Self-adjuvant nanomaterials can enhance the immunogenicity of DNA vaccines through various mechanisms, including (1) Improved antigen delivery: Nanomaterials can protect the DNA vaccine from degradation, facilitate cellular uptake, and enhance antigen presentation to immune cells, leading to increased immune activation. (2) Activation of innate immune response: Some nanomaterials possess innate immune-stimulating properties, such as TLR agonists, which can trigger the activation of immune cells and promote a more robust immune response. (3) Modulation of immune signaling: Nanomaterials can modulate immune signaling pathways, such as the activation of specific cytokines or chemokines, leading to enhanced immune cell recruitment and activation. (4) Co-delivery of multiple components: Nanomaterials can be engineered to co-deliver the DNA vaccine along with other immunostimulatory molecules, such as cytokines or co-stimulatory ligands, to further enhance the immune response.

**Figure 1:**
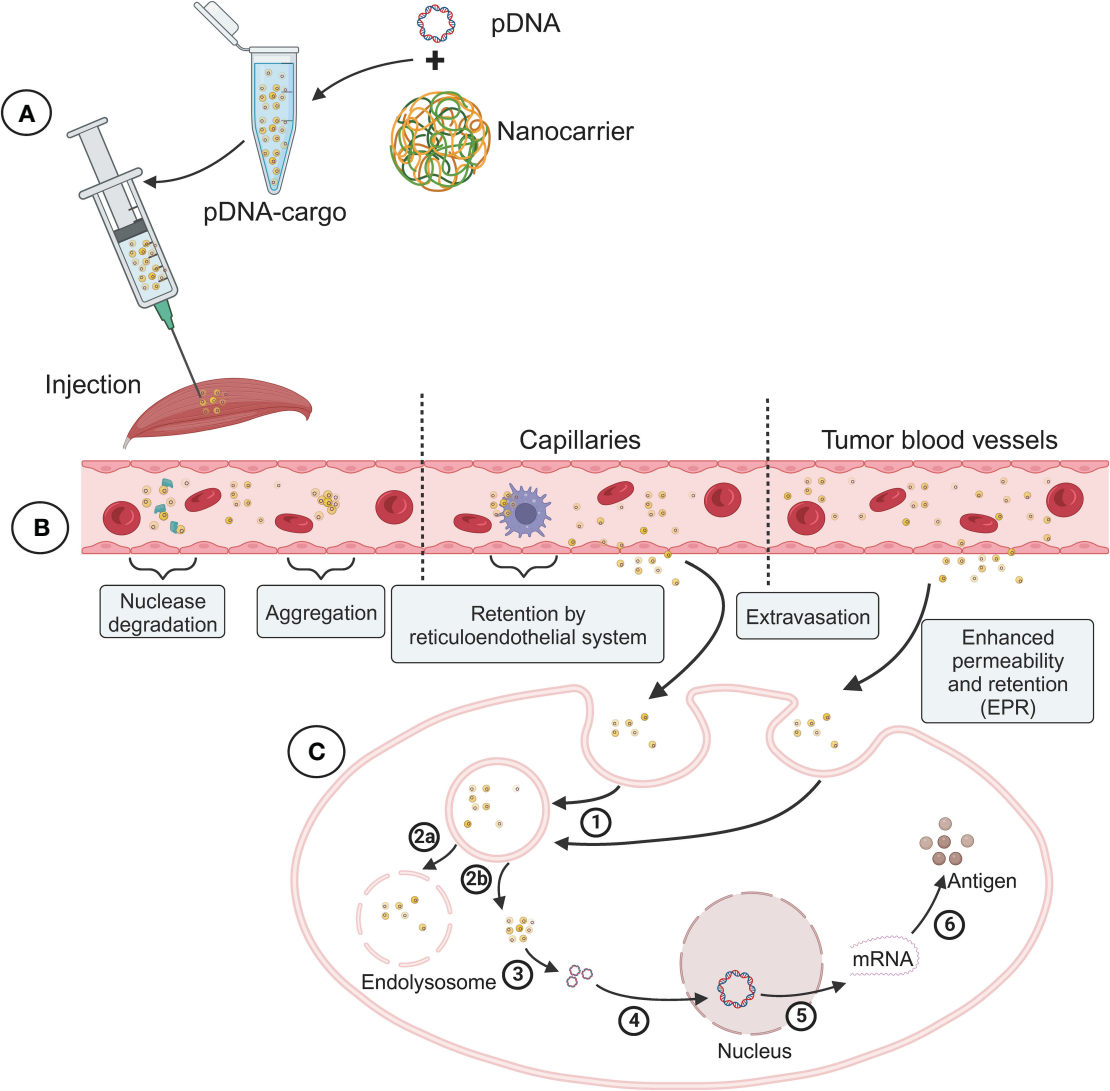
Schematic diagram of the preparation of DNA vaccine nanoparticle complex and its delivery process and challenges *in vivo*. **(A)** Packaging of DNA vaccine nanoparticle complex (pDNA-Cargo). **(B)** Blood circulation and tissue targeting of pDNA-Cargo. **(C)** Intracellular transport and nuclear antigen expression of pDNA-Cargo. 1) Uptake by cell. 2a) Lysosome phagocytosis. 2b) Endosomal escape. 3) DNA release. 4) DNA into the nucleus. 5) Transcription. 6) Translation.

In addition to adjuvants and nanocarrier modifications, the delivery system used for DNA vaccines plays a critical role in their efficacy and the level of immune response. Optimizing the delivery system has been an important strategy in improving the immunogenicity of DNA vaccines and has been the focus of numerous research studies in the past decade. In our review, we also provide a summary of the latest research progress on DNA vaccines in ongoing clinical trials, including information on the different delivery systems being employed (**Table 1**). The primary goal of an effective delivery system is to deliver the antigen protein encoded by the DNA vaccine to the APCs. Thus, the effective delivery system plays a crucial role in both vaccinations and gene therapy (**Figure 1** **).** For instance, nanocarriers can protect DNA from nuclease degradation, ensure its long-term stable circulation in the bloodstream, and facilitate targeted tissue aggregation (40). Additionally, the delivery system helps the DNA escape from endosomes and lysosomes, traverse the nuclear membrane, and other biological barriers to reach the nucleus for transcription. This enhanced immunogenicity promotes APC uptake and subsequently triggers a robust T and B cell immune response. By utilizing effective delivery systems, researchers aim to optimize the delivery of DNA vaccines to maximize their immunogenicity, improve their stability, and enhance the overall immune response. These advancements in delivery system technologies hold great promise for the development of highly effective DNA vaccines and their successful translation into clinical applications (41).

**Table 1:** Genomic material, packaging capacity, tropism, and biosafety level of common viral vectors for DNA vaccines delivery.

Virus type	Genomic material	Packaging capacity	Tropism	Biosafety level
Adenovirus	dsDNA	~30 kb	Broad (dividing and non-dividing)	BSL-2
Adeno-associated virus	ssDNA	~4.8 kb (single AAV)	Broad but distinct serotypes (dividing and non-dividing)	BSL-1
Retrovirus	RNA	~9kb	Broad, dividing (retrovirus and lentivirus) and non-dividing (lentivirus)	BSL-2
Herpes simplex virus (HSV)	dsDNA	~ 160 kb	Preference for neuronal cells, but it still has a broad host and cell type range (dividing and non-dividing)	BSL-2
Hepatitis B virus (HBV)	cccDNA	~ 750bp	Non-dividing cells, distinct tropism (liver),	BSL-2
Baculovirus	dsDNA	~ 38 kb	Infects dividing and non-dividing cells, and are generally used for transducing insects.	BSL-2

**Figure 2:**
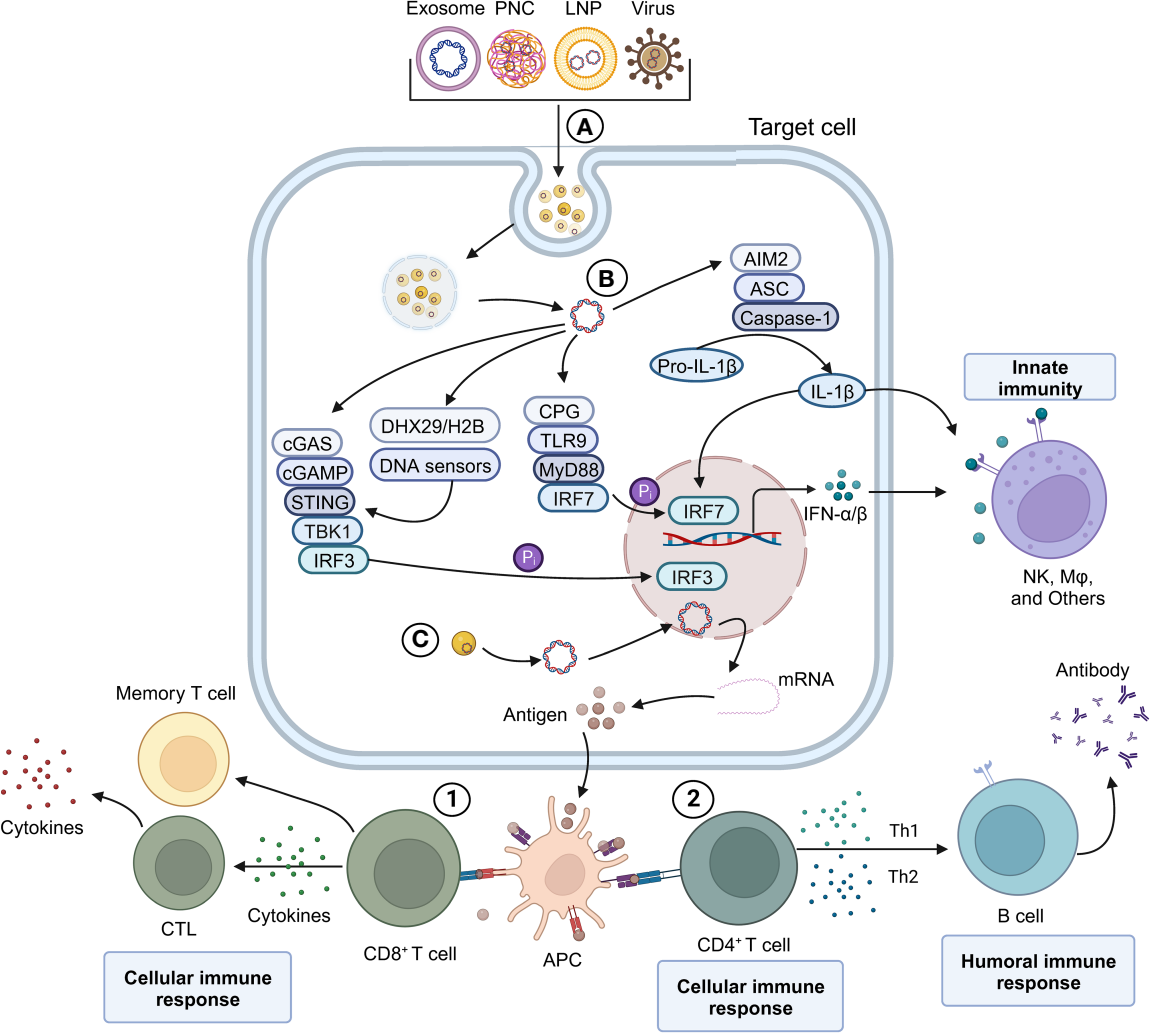
Schematic overview of DNA vaccine mediating innate and adaptive immune response mechanism. **(A)** Various viral and non-viral-based DNA vaccines. **(B)** Plasmid DNA induces the expression of IFN-α/β *in vivo* and activates the IFN-α/β-mediated innate immune response. **(C)** Antigen-presenting cells (APC) process the antigen expressed by pDNA-Cargo and trigger an adaptive immune response mediated by CD8 T and CD4 T cells. 1) CD8 T-mediated cell immune response. 2) B cell-mediated humoral immune response.

The action mechanism of DNA vaccines is illustrated in **Figure 2**, as discussed in this review. The initial step involves the recognition of DNA vaccines by pattern recognition receptors (PRRs) of the innate immune system. This recognition leads to the activation of innate immune responses, including the production of type I interferons and inflammatory cytokines. Several innate immune stimulation pathways associated with DNA vaccines, such as the CpG-TLR9-MyD88 (42, 43), the GMP-AMP (cGAMP)-STING (44, 45), and other DNA sensors such as AIM2 and HMGB1 (23, 46). To trigger a durable adaptive immune response, DNA vaccines rely on the effective presentation of antigens. The DNA is taken up by somatic cells and expressed, leading to the production of antigen proteins. These proteins can be presented to CD8^+^ T cells in the form of major histocompatibility complex (MHC) class I complexes. Additionally, DNA plasmids can express antigens at the injection site, and be directly present to T cells by APCs in the form of both MHC class I and class II complexes. Moreover, specialized APCs can phagocytose somatic cells, which expressing the DNA plasmid, thereby triggering antigen presentation to T cells through cross-presentation (47, 48).

## 3 DNA delivery systems

**Figure 3:**
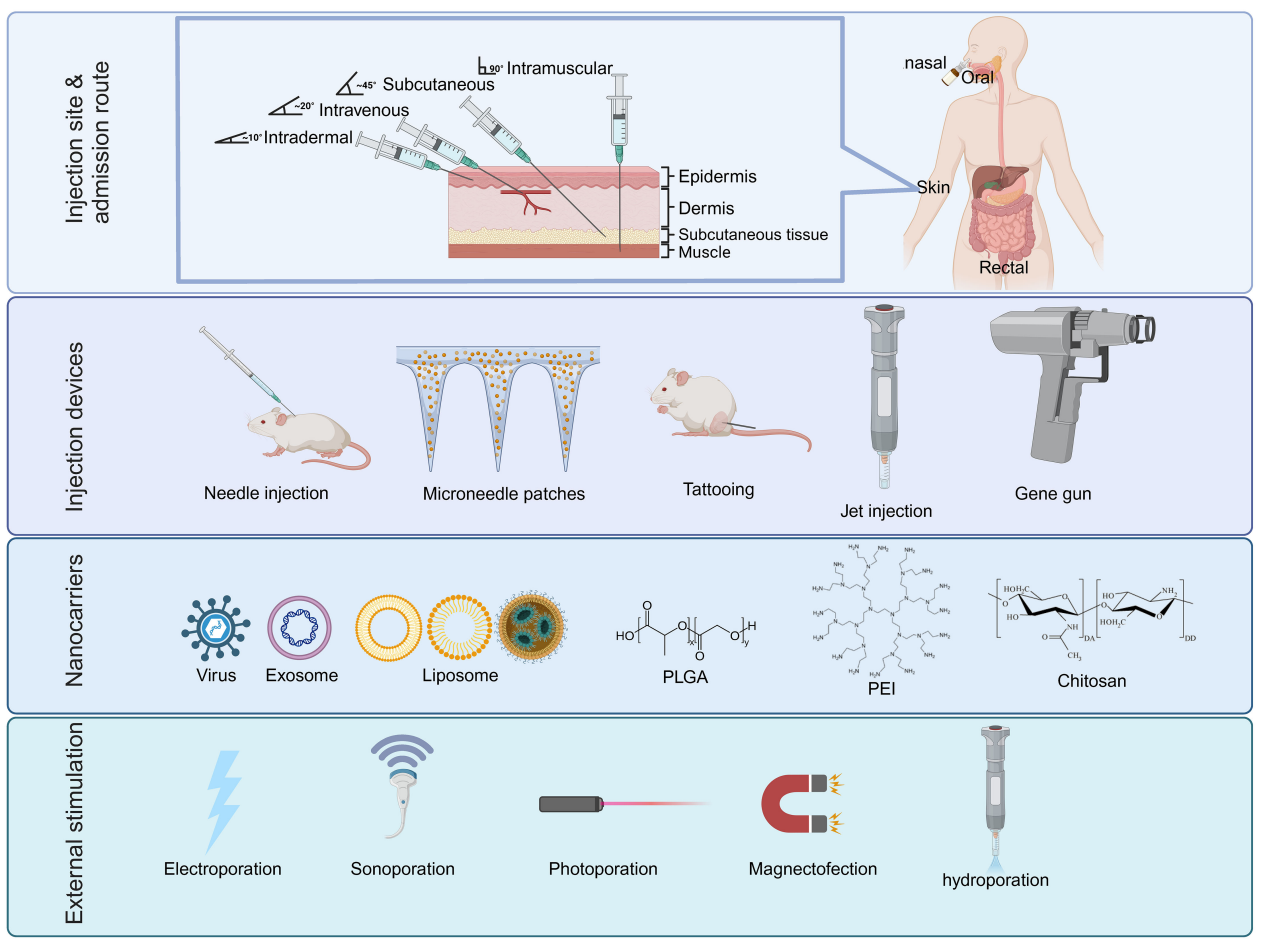
The main components of various DNA delivery systems. DNA vaccines generally have four different components that can enhance immunity through high throughput gene delivery to the target cells: Injection site/admission route, injection instrument, nanocarriers with adjuvants, and external stimulations for temporary cell permeabilization. PLGA, poly D,L-lactic-co-glycolic acid; PEI, Polyethylenimine.

Nucleic acid delivery systems facilitate the transportation of exogenous substances. The delivery system facilitates the target cell delivery followed by passing of the two major membranes (plasma and nuclear) and transports the substance to the nucleus for processing. The delivered DNA is transcribed to mRNA within the nucleus before being exported to the cytosol for translation into the target protein. Host-synthesized antigens expressed by DNA vaccines are therefore capable of eliciting humoral and cellular immune responses. There have been several delivery methods developed, including vector-based (viral), mechanical (microinjection, pressure, particle bombardment), electrical (electroporation), and chemical (ionic- and polymer-based). Next, we will summarize various efforts and research strategies on vaccine delivery systems over the past few decades. These delivery systems have proven successful in delivering DNA to specific tissues or cells to overcome the main challenge of low immunogenicity associated with DNA vaccines (**Figure 3**).

### 3.1 Viral delivery systems

Viral vectors, widely utilized in gene therapy, have emerged in the 1980’s as one of the most common vectors for delivering and expressing exogenous genes. Various viral vectors have been developed for gene delivery, including retrovirus (RV) (49, 50), lentivirus (LV) (51–53), adenovirus (Ad) (54, 55), adeno-associated virus (AAV) (56, 57), and herpes simplex virus (HSV) (58). These vectors exhibit high transfection efficiency and possess inherent mechanism to enter cells and overcome endosomal restrictions, facilitating the delivery of DNA to the cytosol. Additionally, these viruses feature well-organized structures and diverse nuclear localization signal proteins (NLS), enabling efficient recognition of nuclear transport proteins and subsequent DNA delivery to the nucleus for expression. Significant progress has been made in the genomic material, packaging capacity, tropism, and biosafety level of commonly used viral vectors for DNA vaccine delivery (**Table 2**).

**Table 2:** Ongoing clinical trials investigating DNA vaccines.

Clinical Trial Identifier Code	Investigation Plan	DNA Vaccines, Drug/s	Primary Endpoint	Stage of Development	Clinical Trials Status	Delivery Method
NCT04333459	132 participants, Randomized, Parallel Assignment, Double Masking (Participant, Investigator)	Hataan DNA Vaccine/Puumala DNA Vaccine	80% seroconversion	2	Recruiting	The Pharmajet Stratis® Needle-Free Jet Injection Delivery Device
NCT04090528	60 participants, Randomized, Parallel Assignment, Open Label	pTVG-HP, pTVG-AR, Pembrolizumab	PFS	2	Recruiting	pTVG4 vector
NCT03600350	19 participants, Single Group Assignment, Open Label	pTVG-HP, Nivolumab, GM-CSF	Safety, efficacy	2	Active, not recruiting	pTVG4 vector
NCT04397003	27 participants, Non-Randomized, Single Group Assignment, Open Label	Neoantigen DNA vaccine, Durvalumab	Safety, Feasibility of combining durvalumab with a neoantigen vaccine	2	Recruiting	EP (TDS-IM v2.0 Device)
NCT04591184	36 participants, Randomized, Parallel Assignment, Triple Masking (Participant, Investigator, Outcomes Assessor)	Covigenix VAX-001	Safety	1/2	Recruiting	proteo-lipid vehicle (PLV)
NCT05242965	40 participants, Randomized, Sequential Assignment, Single masking (Participant)	CD105/Yb-1/SOX2/CDH3/MDM2-polyepitope Plasmid DNA Vaccine, Sargramostim	Safety, immunogenicity	2	Recruiting	mammalian expression vector pUMVC3 (pNGVL3)
NCT04251117	36 participants, Single group assignment, Open Label	GNOS-PV02, INO-9012, Pembrolizumab	Safety, immunogenicity	1/2	Active, not recruiting	EP (CELLECTRA®2000 EP Device)
NCT04079166	87 participants, Single Group Assignment, Open label	SCIB1 DNA Vaccine	Safety and tolerability	2	Recruiting	PharmaJet Stratis® needle-free injection device system
NCT05455658	33 participants, Single Group Assignment, Open Label	CD105/Yb-1/SOX2/CDH3/MDM2-polyepitope Plasmid DNA Vaccine, Sargramostim	Immune response	2	recruiting	mammalian expression vector pUMVC3 (pNGVL3)
NCT04357821	11 participants, Single Group Assignment, Open Label	Combination Intervention	Safety and efficacy	1/2	Active, not recruiting	MVA
NCT04989946	39 participants, Randomized, Parallel Assignment, Open Label	Degarelix, pTVG-AR, Nivolumab	safety	1/2	Recruiting	pTVG4 vector
NCT03750071	30 participants, Single Group Assignment, Open Label	VXM01, Avelumab	safety and tolerability	1/2	Active, not recruiting	an attenuated strain of the bacterium Salmonella typhimurium
NCT04329065	16 participants, Single Group Assignment, Open Label	pUMVC3-IGFBP2-HER2-IGF1R Plasmid DNA Vaccine, Paclitaxel, Trastuzumab, Pertuzumab	Immune response	2	Recruiting	mammalian expression vector pUMVC3
NCT03439085	77 participants, Single Group Assignment, Open Label	DNA Plasmid-encoding Interleukin-12/HPV DNA Plasmids Therapeutic Vaccine MEDI0457, Durvalumab	Immune response	2	Active, not recruiting	EP
NCT04066881	1668 participants, Randomized, Parallel Assignment, Triple Masking (Care Provider, Investigator, Outcomes Assessor)	DNA-HIV-PT123, AIDSVAX® B/E, CN54gp140+MPLA-L, MVA, TAF/FTC, TDF/FTC	safety	2	Enrolling by invitation	MVA
NCT03603808	80 participants, Single Group Assignment, Open Label	HPV DNA Plasmids Therapeutic Vaccine VGX-3100	effectiveness	2	Active, not recruiting	Electroporation
NCT05141721	665 participants, Randomized, Parallel Assignment, Open Label	GRT-C901, GRT-R902, Atezolizumab, Ipilimumab, Fluoropyrimidine, Bevacizumab, Oxaliplatin	effectiveness	2/3	Active, not recruiting	chimpanzee adenovirus vector (ChAdV)
NCT05334706	69 participants, Single Group Assignment, Open Label	Nonavalent HPV vaccine (9vHPV/Gardasil-9™)	efficacy	2	Recruiting	Virus-like Particle
NCT05099965	90 participants, Randomized, Parallel Assignment, Double Masking (Participant, Investigator)	CMV-MVA Triplex	safety and immunogenicity	2	Recruiting	MVA
NCT03911076	134 participants, Randomized, Parallel Assignment, Quadruple Masking (Participant, Care Provider, Investigator, Outcomes Assessor)	PVX-2 [pNGVL4a-Sig/E7(detox)/HSP70 (naked DNA plasmid priming vaccine)]	Safety and efficacy	2	Recruiting	viral vector (Vaccinia virus)
NCT04778904	52 participants, Randomized, Sequential Assignment, Open Label	ChAdOx1-HBV, MVA-HBV, Nivolumab	safety	1/2	Active, not recruiting	chimpanzee adenovirus (ChAd) and modified vaccinia Ankara (MVA) viral vectors
NCT04983030	36 participants, Randomized, Parallel Assignment, Double Masking (Participant, Investigator)	Ad26.Mos4.HIV, MVA-BN-HIV, PGT121, PGDM1400, VRC07-523LS	Safety, Immunogenicity/efficacy	1/2	Recruiting	Adenovirus, Modified Vaccinia Ankara - Bavarian Nordic (MVA-BN®) vector
NCT02285816	56 participants, Non-Randomized, Parallel Assignment, Open Label	MG1MA3, AdMA3	Safety and efficacy	1/2	Active, not recruiting	Adenovirus
NCT03866187	148 participants, Randomized, Sequential Assignment, Single Masking (Participant)	ChAd155-hIi-HBV, HBc-HBs/AS01B-4, MVA-HBV	safety	1/2	Active, not recruiting	Adenovirus, Modified Vaccinia Ankara
NCT04607850	105 participants, Randomized, Sequential Assignment, Quadruple Masking (Participant, Care Provider, Investigator, Outcomes Assessor)	ChAdOx1-HPV, MVA-HPV	safety	1/2	Active, not recruiting	Adenovirus, MVA
NCT05904054	60 participants, Non-Randomized, Parallel Assignment, Open Label	SARS-CoV-2 DNA Vaccine (ICCOV)	efficacy	2	Recruiting	MVA-BN
NCT02157051	42 participants, Non-Randomized, Sequential Assignment, Open Label	CD105/Yb-1/SOX2/CDH3/MDM2-polyepitope Plasmid DNA Vaccine	safety	1	Active, not recruiting	Intradermal injection
NCT04090528	60 participants, Randomized, Parallel Assignment, Open Label	pTVG-HP/ pTVG-AR DNA vaccine, Pembrolizumab	safety	2	Recruiting	Intradermal injection, intravenous injection
NCT05905354	12 participants, Sequential Assignment, 3+3 dose climb, Open Label	DNA vaccine NWRD08	safety	Not Applicable	Recruiting	Electric pulse gene delivery instrument
NCT04131413	48 participants, Non-Randomized, Sequential Assignment, Open Label	pNGVL4aCRTE6E7L2 DNA vaccine	Safety, Tolerability, and Feasibility	1	Recruiting	Intramuscular TriGridTM Electroporation Delivery System
NCT06002503	40 participants, Randomized, Parallel Assignment, Double (Participant, Investigator)	Venezuelan Equine Encephalitis DNA Vaccine,	Safety, Reactogenicity and Immunogenicity	1	Recruiting	Intramuscular (PharmaJet Stratis Needle-free Injection System)
NCT05743595	12 participants, Non-Randomized, Sequential Assignment, Open Label	Personalized Neoantigen DNA vaccine, Retifanlimab	safety	1	Recruiting	TDS-IM v 2.0 electroporation device
NCT06046092	27 participants, Sequential Assignment, Open label, single arm dose escalation phase I trial, Open Label	H7HLAII DNA vaccine	safety	1	Recruiting	Intradermal injection
NCT06088459	9 participants, Sequential Assignment, 3+3 dose escalation principle, Open Label	NWRD06 DNA vaccine	safety and immunogenicity	1	Recruiting	Electroporation

#### 3.1.1 Adenovirus

Adenovirus (Ad) is a non-enveloped microbe characterized by an icosahedral coat protein and a double-stranded DNA genome ranging from 26 to 45 kb in length, with a diameter of approximately 90 nm (59). Upon entering a host cell, Ad type 5 (Ad5) initially expresses the “early-phase” genes, which include E1A, E1B, E2, E3, and E4 located between the inverted terminal repeats (ITR) sequences. These genes are involved in viral replication (60). Alterations or replacements of these E genes can modify the virus’s replication and enable the engineering of recombinant Ads for gene therapy. Cells are infected through the binding of Ad fibers to Ad receptors. The viral capsid binds to the nuclear pore complex near the nuclear membrane pore and with the assistance of dynein, the capsid is disassembled, allowing the viral genome to enter the nucleus for target gene expression (61, 62). Ad has the capacity to package and deliver fragments of approximately 37 kb in size. It efficiently transduces a wide range of target cells, including both dividing and quiescent cells. To minimize risks, the integration of viral genomic DNA into the host genome is tightly regulated by specific knockout or recombination of the “early phase” genes (63).

Recombinant Ads (rAds) encompass various types, including conditionally replicating Ad (CRAd), replication-defective Ad (RDAd), and helper-dependent Ad (HDAd). These vectors have gained popularity in the fields of cancer and regenerative medicine (64), particularly in promoting anti-tumor immune responses through the introduction of tumor-associated antigens (TAA) (35, 65). CRAd, also known as oncolytic Ad, is specifically designed to target cancer cells and release the TAAs due to its high cytotoxicity, making it a potent tool in combating tumors. On the other hand, most Ad vectors used are replication-defective Ads, as the safety of some of these vectors has been verified through clinical trials (66). One successful example is the modified chimpanzee adenovirus-vectored vaccine(ChAdOx1), which has demonstrated robust and long-lasting immune responses against various diseases, including but not limited to Covid-19(Oxford-AstraZeneca vaccine), Zika virus, prostate cancer, influenza and malaria. This highlights the sustained cellular and humoral responses achieved with this vector (67). Currently, researchers are exploring its potential for wider applications in challenging new therapeutic and prophylactic vaccines through preclinical and clinical trials. Notably, both Oxford-AstraZeneca and Johnson & Johnson Covid-19 vaccines have showed a low risk of developing thrombosis, specifically, thrombocytopenia syndrome (TTS) (68). Furthermore, HDAd, often referred to as high-capacity Ad due to its ability to accommodate large cargo sequences of up to 37 kb (69), involves the removal of all viral genes from the vector backbone except for the ITR, and wild-type Ad packaging signals. This enables long-term gene expression without causing chronic toxicity (70). For instance, Tanoue et al. successfully used HDAd to express the PD-L1 antibody without toxicity from infection, effectively blocking the PD-1 to PD-L1 interaction (71). In addition to cancer therapies, HDAd has shown potential in restoring cellular function affected by a nonsense mutation to a healthy control range, as demonstrated by the CFTR gene-encoded HDAd (72).

#### 3.1.2 Recombinant adeno-associated virus

Recombinant adeno-associated virus (rAAV) is a non-enveloped single-stranded DNA virus belonging to the Parvoviridae family. Its natural coding and non-coding regions have been replaced with an expression cassette of approximately 4.8 kb. This cassette is flanked by two T-shaped ITRs and contains the *Rep* (Replication) and *Cap* (Capsid) genes (73). AAV infection occurs through receptor-mediated endocytosis, where the capsid is transported into the nucleus via an endosome. Once inside the nucleus, the internalized single-stranded DNA undergoes replication to form a complementary strand, increasing stability (57). To overcome the cargo size limitation, innovative approaches involve splitting the transgene into two or more separate rAAV vectors. This trend has led to the development of triple AAV, which can accommodate a cargo size of up to 14 kb, as the ITR allows trans-splicing of pre-mRNA (74, 75). Another method for increasing cargo size involves utilizing intein-mediated protein trans-splicing (PTS). This approach is actively being investigated in clinical studies of lung gene therapy for treating hemophilia A (76). Esposito et al, employ PTS to shuttle factor VIII using two separate single-stranded DNAs coding for N- and C- inteins, which connect to half of the coding sequence. After translation and PTS, they achieve the expression of the full-length therapeutic factor VIII-N6 variant (77). rAAV is generally considered non-pathogenic and exhibits low toxicity. Similarly, to Ad, the free DNA nature of rAAV is associated with lower mutagenicity. Despite its broad tissue tropism, each tissue type requires a specific serotype for efficient transduction (78). Currently, rAAV is widely used in gene replacement to test new therapies in animal models of diseases (79), evaluate gene function (80), and knock out gene expression (81).

#### 3.1.3 Retroviruses and lentiviruses

Retroviruses (RV) is a single-stranded RNA virus with a capsid and envelope, typically ranging from 80 to 120 nm in diameter. The RV genome contains several essential genes, including *Gag* (encoding structural protein), *Pol* (encoding reverse transcriptase and integrase), and *Env* (envelope protein for attachment protein). These genes are flanked by enhancers and promoter long terminal repeats (LTR) (82). RVs have the capability to package and deliver genes of up to 9 kb in size, exhibit a wide range of tropism, and can achieve long-term transgenic expression. However, one of the main disadvantages of RV vectors is the potential for insertional mutagenesis at the integration site. This can occur due to the disruption or inappropriate activation of nearby host gene’s transcription (83). Integration into regulatory elements such as enhancers leads to adverse consequences (84). RVs utilize positive-sense single-stranded RNA as a template to reverse transcriptase enzyme. This viral DNA is then integrated into the host genome. Multiple RVs can serve as vectors for gene delivery, including lentivirus (LV), spumavirus, and gammaretrovirus (85). While most RVs can only infect dividing cells, LV is the exception and is capable of infecting both dividing and non-dividing cells. Currently, RVs, particularly LVs, are widely used as viral vectors in the design of chimeric antigen receptor CAR-T cells (86). Therefore, RVs indeed considered a highly effective viral delivery method.

LVs, a genus of the RV family, retroviruses have been extensively studied as a vector, particularly HIV vectors. LVs have the ability to integrate into the host genome and have been employed in various treatments. Some of the latest applications of LVs include the treatment of chronic granulomatous disease (87), sickle cell disease (88), Wiskott-Aldrich syndrome (89), atherosclerosis (90), severe combined immunodeficiency (SCID) (91), and human immunodeficiency virus (HIV) (92). Compared to other RVs such as gammaretrovirus, LVs have the advantage of being able to infect non-dividing cells, thanks to the presence of proteins with nuclear localization signals (93). LVs utilize a plasmid with a long terminal repeat (LTR) promoter for gene expression in the second-generation LV vectors, with most accessory genes removed except for Tat and Rev (94). These vectors are often pseudotyped, typically using the vesicular stomatitis virus G protein (VSV-G), to enhance safety by preventing the formation of a replicative virus (95, 96). In the third-generation LV vectors, the *Rev* gene is separated from the plasmid containing the *Gag* and *Pol* genes. The LTR is modified by removing enhancer and promoter regions, resulting in a self-inactivating (SIN) vector that is replicating-incompetent and less oncogenic (96). LV vectors have tremendous potential due to their ability to transduce quiescent cells, including DCs, which are highly effective in presenting antigens. Transduction of DCs with LV vectors has been shown to yield strong efficiency, as demonstrated by GFP expression in rat and mouse DCs (97). The SIN design of LV vectors can also be applied to other retrovirus. For example, in gamma RVs, such as Moloney murine leukemia virus (MLV), SIN gammaretrovirus corrected the gene *IL2RG* mutation that resulted in SCID-X1 on hematopoietic stem cells. These vectors have shown promising results in terms of T-cell reconstitution and clearance of viral infections (98). In immunotherapy, MLV has been derived to enhance safety and optimize gene expression, leading to the development of MFG and SFG vectors (99, 100). Foamy virus, a member of the spumavirus family, and is considered nonpathogenic, making it a safer option for gene delivery compared to other RVs. Studies have demonstrated its efficacy *in vivo* for delivering genes in the treatment of canine SCID-X1, with over 75% of lymphocytes undergo reconstitution. Although off-target transductions are low, they are still present (101). Overall, foamy virus vectors offer a potentially safe and effective gene therapy approach. Due to concerns about genetic integration, RVs are still commonly used in *ex vivo* studies of clinical trials.

While AAV, Ads, and RVs have been leading the race in viral vectors, other viruses like herpes simplex virus (HSV), hepatitis B virus, and baculovirus, are also actively being investigated for their potential in gene delivery. In short, viral vectors hold promise as effective delivery vehicles for introducing genes into cells. Their ability to naturally deliver genes into the nucleus, facilitating efficient gene expression, sets them apart from non-viral vectors. However, viral vectors can elicit immune responses and carry the risk of integrating into the host genome. To address these concerns, researchers have made significant advancements in molecular manipulation techniques, including pseudotyping, self-inactivation, and gene elimination, to reduce or avoid side effects. These advancements have enabled successful applications of gene therapy in animal models and have positioned viral vectors as more refined gene delivery tools.

### 3.2 Non-viral delivery systems

Non-viral delivery systems have made significant advancements in the past decade. These systems primarily rely on nanoparticles (NPs), which have a broad impact in various biotechnological and medical fields, including targeted therapies or diagnostic tools for the treatment of peripheral arterial disease (102, 103). NPs-based gene delivery utilizes recombinant DNA technology and nano-synthesis technology to deliver DNA to cells *in vivo* and *in vitro* as an alternative treatment for various primary or secondary diseases, such as genetic diseases (104), cancer (105–107), cardiovascular diseases (108), and immune system diseases (109–112). However, the success of gene therapy encounters several challenges. These include rapid degradation of plasmid DNA by nuclease, limited targeting ability and poor uptake of target cells, difficulties in nucleation and low transfection efficiency, limited transfection capacity, and immune responses of immune cells. As a crucial component of non-viral vector delivery system, gene delivery using NPs can encapsulate DNA therapeutics through electrostatic interaction or chemical bonding. This encapsulation significantly enhances the biological characteristics, kinetic properties, and therapeutic efficacy of the embedded DNA therapeutics, thereby overcoming the various challenges encountered in gene therapy (113, 114). NPs can safeguard DNA from degradation by nucleases and prolong the circulating half-life (115). Furthermore, nanoparticles can interact with immune receptors and proteins, thus disrupting signaling cascades (116). NPs can be modified by targeting ligands and molecules to promote the nuclear uptake by cells expressing the target protein. This modification improve delivery efficiency and reduces toxicity (117). Additionally, personalized modifications significantly enhance the ability of NPs to carry larger DNA sizes and offer flexibility for specific cell types, particularly in the context of gene delivery of cancer DNA vaccines. NPs can deliver nano-DNA vaccines to cancer cells more effectively, minimizing the risk of damage to normal tissues. This capability is advantageous for immunotherapy against solid tumors (118, 119). ​​Additionally, NPs used in cancer DNA vaccines can be modified to respond to the endosomal environment, such as the acidic microenvironment of tumors. This modification allows for control over the degradation and release of nucleic acids within the NPs, thereby manipulating the immune response (120).

The rapid development of nanotechnology has enabled NP delivery systems to meet the demanding requirements in applications and clinical research, including disease monitoring, imaging, DNA drug delivery, and treatment (121–123). NPs have particularly emerged as the most commonly used, safe, and effective delivery platform for gene therapy compared to traditional delivery vehicles. They have become the preferred delivery platform for most cancer DNA vaccines. The use of NPs in conjunction with DNA vaccines enhances DNA delivery, improves transfection efficiency, reduces toxicity, and promotes targeting and immune response.

### 3.3 Cationic delivery vehicle

#### 3.3.1 Liposomes

Liposomes are spherical or near-spherical micro-vesicles composed of a lipid bilayer consisting of phospholipids and sterols. The hydrophilic side wraps the water phase inwardly, and the hydrophobic end is suspended in the aqueous medium. According to the principle of similarity and compatibility of biofilms, it can be fused with cell membranes; since then, liposomes and liposome-derived nanovesicles have been used as delivery system carriers in medicine and pharmacy (124, 125). The main advantages of liposome-based delivery systems are their multifunctionality and versatility. They can effectively encapsulate both hydrophilic and hydrophobic compounds, such as proteins, lipopeptides, and other therapeutic agents (126). Through adsorption or stable chemical bonds or electrostatic binding, antigens, nucleic acids molecular substances are attached to the surface of liposomes to achieve the required characteristics and even targeting functions (127–129).

Cationic liposomes can form complexes with negatively charged DNA through natural electrostatic binding. As a result, cationic liposomes are suitable transfection reagents and are widely used as DNA vaccine carriers for mammalian cells (130, 131). Various parameters influence the delivery efficiency of liposomal DNA complexes in the body, including liposome size, cationic lipid composition, and surface charge density of liposomal DNA complexes (132–134). Furthermore, the ability of liposomes to deliver the protein encoded by DNA triggers a robust T-cell and antibody response (135). Liposomes with high charge density have been shown to enhance the maturation of DCs, the production of reactive oxygen species (ROS), the uptake of antigens, and the production of IgG2a and IFN-γ, effectively promoting the immune response (136, 137). The most commonly used cationic liposomes for nucleic acid delivery are 1,2-dioleoyl-3-trimethylammonium-propane (DOTAP) (138) and N-[1-(2,3-dioleyloxy) propyl-N,N,N-trimethylammonium chloride (DOTMA) (139). These lipids are highly flexible and exhibit high drug retention for nucleic acids due to their opposing electrochemical charge. Additionally, research in our laboratory has shown that ALC-0315 or SM-102 cationic liposomes can efficiently deliver DNA and demonstrate good expression efficiency, making them promising tools for develop DNA vaccines or gene therapies in the future. Compared to neutral liposomes, cationic liposomes can better encapsulate the drug and facilitate the release of nucleic acid in cells. The integration of an appropriate level of cholesterol further enhances their capabilities (140). The application of cationic liposomes extends to pH-sensitive drug carrier and cancer drug delivery. The acidic microenvironment in cancer cells can destabilize the pH-sensitive carrier’s membrane, releasing the drug at the appropriate time and location (141). However, cationic lipid carriers often have a short half-life, and PEGylation is a common approach to extend their circulation half-life and increase immunity evasion. On the other hand, the host can develop an adaptive immune response, producing anti-PEG immunoglobulins that leading to accelerated blood clearance during subsequent immunization with nucleic acid vaccines (142, 143). An alternative to the modification is the use of 1,2-dioleoyl-sn-glycero-3-phosphoethanolamine (DOPE), is a widely used helper lipid that can enhance the transfection efficiency. However, it may present high toxicity at the required amount of lipid for nucleic acid delivery (144).

Despite these challenges, the versatility and plasticity of liposomes make them an invaluable delivery system for DNA vaccines. Liposomes can protect DNA, enhance its stability, and serve as a potent immune adjuvant, stimulating the activity of the immune system and inducing an antigen-specific immune response. Their compatibility characteristics enable them to overcome biological barriers, making liposomes a promising gene delivery method that provides important insights for vaccine development.

#### 3.3.2 Cationic polymer: polyethyleneimine

PEI is a cationic polymer with abundant amino groups that can be utilized as an effective delivery system. It possesses the following characteristics: 1) The high-density amino cations allow for strong electrostatic interaction and polymerization of nucleic acids, forming multi-stranded complexes that can protect nucleic acids from degradation by ribozymes. 2) The interaction between the positively charged polymer and the cell surface promotes cellular uptake and internalization, and the high amine density facilitates the entry of multimers into the nucleus from the lysosomal compartment for transcriptional expression. Currently, PEI is considered the most promising cationic carrier and is capable of achieving high transfection efficiency in various mammalian cells (145). PEI exhibits the “proton sponge” effect and possesses excellent buffering capacity at acidic pH, allowing it to escape DNA molecules from lysosomal compartment, making it an ideal carrier for gene therapy (146–148). In a recent study comparing PEI to anionic, neutral liposomes, DOTAP, and chitosan for intramuscular and intranasal vaccination, PEI demonstrated superior antibody responses (IgG1 and IgG2), inflammatory cytokines, DC maturation, and cellular immunity (149). PEI is also used for the delivery of cancer gene vaccines. For example, the PEI/DNA complex, PEI/pAc-neo-OVA, has shown efficacy in treating the mouse EG7-OVA thymoma model by inducing protective and therapeutic immunity and prolonging mouse survival (150). In the field of anti-infective vaccines, intranasal co-administration of PEI/M2 (M2e) complex induces a systemic and mucosal humoral immune response against M2e, significantly increasing antibody levels (IgG, IgA) and CD4^+^ T cell response, thereby protecting chickens from H9N2 influenza A virus infection (151). However, despite its advantages as a DNA vaccine delivery system, PEI faces limitations in delivering plasmid DNA therapy due to its high cytotoxicity. The strong electrostatic interaction of high molecular weight (MW) PEI with cell membranes and extracellular matrix can cause severe cytotoxicity and reduce blood compatibility (152). This can lead to endosome swelling, rupture, intracellular stress, mitochondrial changes, ultimately cell death (153). Cytotoxicity increases with the number of branches and charge density. PEI with MW between 5 and 25 kDa achieves the best transfection efficiency with the low toxicity, but it has a short half-life, short blood circulation time, and limited ability to induce lasting immunity. Furthermore, its biocompatibility is poor, and repeated administration increases the risk of toxicity. To address these issues, researchers have incorporated biocompatible components such as polyethylene glycol (PEG) (154), PLGA (155), and cyclodextrin (156) to modify PEI, aiming to reduce its cytotoxicity, extend blood circulation time, and trigger a sustained immune response. Other cationic polymers can also co-deliver DNA, reducing toxicity and increasing transfection efficiency. For example, the formation of a PEI/chitosan/DNA complex with chitosan significantly increases transfection efficiency while exhibiting low cytotoxicity (157, 158).

In conclusion, PEI plays a significant role in the non-viral vector delivery system for gene therapy, particularly as a DNA vaccine delivery carrier. Although there have been limited achievements thus far extensive research has been conducted to explore the potential of PEI in combination with various advantages it offers, such as high transfection efficiency and cationic polymerization. Many composite delivery systems based on PEI have been designed to address challenges related to toxicity, blood circulation, biocompatibility, and controlled-release targeted delivery *in vivo*. These advancements aim to enhance the efficacy of gene therapy and establish PEI as a potentially safe and effective vehicle for DNA delivery.

#### 3.3.3 Chitosan

Chitosan, a (1,4)-2-amino-2-deoxy-D-glucan, is derived from the deacetylation of chitin, a natural biological polysaccharide found in crustaceans. Chitosan possesses several positive properties that make it attractive for gene therapy applications, including biocompatibility, easy of mass production, high biodegradability, good mucosal adhesion, and non-toxicity (159). Chitosan is naturally positively charged and can induce natural adsorption with negatively charged cell membranes or plasmid DNA. This property enhances cell adhesion and absorption, promotes intercellular transport, enables sustained release, and facilitates biodegradability. As a result, chitosan has found widespread used in pharmaceutical excipients and sustained-release agents.

Unmodified chitosan has limitations in physiological conditions due to its solubility. Hydrophilic modification of chitosan with polymers such as PEG can reduce the zeta potential and NP’s size (160). PEG increases repulsion during cellular uptake and endosomal escape by decreasing the NP charge. However, excessively high PEG concentrations may decrease the transfection efficiency (161). The correlation of PEGylation and transfection efficiency is non-linear and requires optimization of multiple variables, including chitosan MW, N/P ratio, PEGylation magnitude, and crosslinking magnitude. In terms of solubility, high MW chitosan polymers have a hydrophobic backbone, which decreases internalization (162). Meanwhile, higher MW chitosan provides better protection and stability for DNA, although it hinders the release of DNA due to larger charge differences between the NP and DNA (163, 164). Chitosan is typically soluble only in acidic environments with a pH below 6, which has limited its success in clinical trials. Therefore, modification of chitosan’s solubility by oxidizing the -OH and -NH group can improve its performance as a biopolymer (162, 164).

Like polycationic PEI, chitosan can condense DNA strands and form compact positive nanoparticles. These nanoparticles can adhere to the cell surface, facilitate endocytosis and internalization, and exhibit robust buffering capacity within the endosomal pH range (pH 4.5 to 7.5). This buffering capacity inhibits the degradation of DNA by lysosomal enzymes and promotes DNA delivery into the nucleus through the “proton sponge” effect, thereby regulating gene expression (165). Compared to the PEI/DNA complex, the chitosan/DNA polyelectrolyte complex has unique physical and chemical properties. It offers biocompatibility and biodegradability recognized safety, non-toxicity, and improved stability. Several parameters influence the formation and properties of the chitosan/DNA complex, including chitosan MW, plasmid DNA concentration, charge ratio (N+/P-, N is the moles of chitosan total -NH2, P is the DNA Phosphate-PO3 moles), and external salt concentration. Studies have demonstrated that the size of particles formed by chitosan/DNA complex decreases with molar mass. Conversely, higher MW enhances the stability of the chitosan/DNA complex, making it more resistant to resist salt and serum. Chitosan has a weakly alkaline pKa of 6.5, which allows it to control the protonation level of amino groups. This control is crucial for maintaining the stability and condensation strength of the complex formed between the positive charge (-N+) and the highly negatively charged DNA (P-). The degree of protonation of amino groups (-NH3+) depends on factors, such as pH, N+/P-ratio (N/P ratio, that is, chitosan unit/phosphate unit), and external salt concentration (166, 167). Therefore, when utilizing chitosan for DNA vaccine delivery, it is essential to achieve an appropriate nanometer size and positive charge to facilitate cell membrane function, internalization, and promote gene expression. Chitosan’s immune adjuvant properties are also widely observed in chitosan/DNA complex vaccine therapy. Chitosan-encapsulated DNA vaccine can effectively induce both mucosal immune response and systemic immune response without causing toxic side effects (168). Chitosan facilitates the maturation of DCs by inducing IFN and enhances the antigen-specific T helper 1 (Th1) response in a manner dependent on the type I IFN receptor (24). By designing a DNA vaccine with mannosylated chitosan to target airway antigens to APCs in the alveoli, it successfully induces multifunctional CD4^+^ T cell responses, leading to increased secretion of TNF-α, IL-2 and IFN-γ (168). Furthermore, the chitosan HPV-16 E7 DNA vaccine significantly enhances specific lymphocyte proliferation index and cytotoxic T lymphocytes (CTL) activity against E7 protein, resulting in higher levels of IFN-γ and IL-4 production and reduced IL-10 production, which promotes tumor regression (169).

As a natural renewable biopolymer, chitosan possesses favorable physicochemical properties as a carrier. It leverages its biocompatibility, degradability, and cationic properties to form complexes with plasmid DNA. By preparing suitable deacetylated chitosan molecules and employing appropriate chemical modifications, as well as controlling factors, such as proper N/P ratio, pH, and external salt concentration, it is possible to overcome the challenge of relatively low transfection efficiency. Consequently, chitosan has found widespread application in various animal models for gene therapy. Moreover, chitosan gel has been employed in clinical settings for tissue repair, while there is still a considerable distance to cover before chitosan-based DNA vaccines can be employed in humans. Chitosan holds great potential as an excellent and effective carrier system for gene therapy.

### 3.4 Anionic polymer: D, L-lactide-co-glycolic acid

PLGA is a negatively charged biocompatible copolymer synthesized from polyglycolic acid (PGA) and polylactic acid (PLA) monomers through condensation polymerization and ring-opening polymerization. Unlike PEI, PLGA has a longer half-life, potential miscibility with specific compounds, and lower cytotoxicity. The polymer can be metabolized through the Kreb’s cycle, producing carbon dioxide and water. The physical and chemical properties of PLGA are influenced by the molar ratio of PGA and PLA as well as the synthesis method (170). Due to its high biocompatibility and tunable biodegradability, PLGA has been extensively utilized in single drug delivery (171), combined cancer immunotherapy (172), and as a biomaterial for tissue engineering and regeneration (173). Various methods are employed to create PLGA, with single and double emulsification being the most common. Each method is suitable for encapsulating hydrophobic and hydrophilic drug molecules. When precise control over drug loading, carrier size, and uniformity is desired, microfluidic mixing of generally immiscible compounds is the recommended approach (174).

The main advantages of PLGA, which include biocompatibility and degradability, good stability, long half-life of blood circulation, controlled release of biologically active molecules, and protection from by endonuclease degradation, have positioned it as an increasingly preferred polymer for DNA vaccine nano-delivery platforms. In the past decade, PLGA microspheres have been formulated using a solid emulsion in oil-in-water (S/O/W) method, with a nano-level core composed of polyethylene glycol/polyethyleneimine (PEG-g-PEI)/pDNA composite. This delivery system has been used to successfully deliver HIV genes, inducing protective humoral and cellular immune responses in mice (99). Additionally, a double emulsion solvent evaporation method has been employed to prepare PLGA nanoparticles (PLGA/pIFN-λ1) containing a plasmid encoding IFN-λ1 (pIFN-λ1), which effectively protects the plasmid DNA from nuclease degradation and enhances gene delivery efficiency to HEK293T cells. As a result, IFN-λ1 expression is achieved and Hep2-C cells are successfully protected against EMCV cells (175). Moreover, PLGA has been utilized as a delivery system for the encapsulation of plasmid DNA encoding Cas9. This system induces the expression of bacterial Cas9 in mouse bone marrow-derived macrophages (BMDMs) *in vitro*, presenting a promising prospection for future applications of the CRISPR-Cas9 system (176). Furthermore, PLGA nanoparticles (PLGA/pcSip) encapsulating the plasmid pcSip, encoding the surface immunogenic protein Sip of streptococcus agalactiae, have successfully expressed Sip in immunized tilapia tissues, providing protection against streptococcal infection (177).

PLGA is an ideal choice for DNA vaccine therapy delivery vehicle, but it faces challenges, such as low DNA encapsulation efficiency and slow DNA release. The negative charges associated with PLGA can hinder the internalization of DNA in cells, DNA release into the nucleus, and expression of delivery genes. Additionally, high shear stress and ultrasonic DNA fragmentation during nanoparticle preparation can lead to DNA inactivation (178). To address these issues, researchers have explored different manufacturing methods. Nanoprecipitation, which involves the use of miscible organic and aqueous solvents to induce spontaneous phase separation by introducing a strong anti-solvent, is a method that does not require intensive shearing rates, high temperature, and ultrasonication. In combination with microfluidics, molecular diffusion of the two solvents can occur more efficiently at the junctions of microchannels, resulting in the formation of uniformly sized nanoparticles. López-Royo et al. compare nanoprecipitation to microfluidics-assisted double emulsion solvent evaporation with polyvinyl alcohol (PVA), and they find that nanoprecipitation had slightly lower entrapment efficiency, with a difference of only 6% (178). The significant progress is being made with larger plasmids, especially using the nanoprecipitation technology. Jo reports a high entrapment efficiency of approximately 80% of ~8.5kb CRISPR plasmid using amine-end capped PLGA (176).

Researchers are also exploring alternatives to ensure high encapsulation efficiency while maintaining the integrity of nucleic acid sequences. One approach involves the use of polyplexes, where the DNA-containing nanoparticle is enveloped within the PGLA microsphere. Although most of the currently used polyplexes are not full biocompatible, they allow biomaterial engineers to balance the equation of cytotoxicity, gene stability, and gene delivery efficiency. While PEI/PLGA (polyethyleneimine modification) is the most commonly used modification technique, other polyplexes offer potential alternatives with superior stability and biocompatibility benefits. PEI has a strong natural highly condensed nucleic acid and the ability to promote DNA expression in the nucleus. The formation of PEI/PLGA copolymer nanoparticles enhances gene delivery efficiency, improves transfection to phagocytes, and enhances serum compatibility (179, 180). However, the efficiency and concentration of specific modifications require comprehensive studies. For instance, the transfection and encapsulation efficiency of PLGA-PEI-PEG-folic acid are higher than those of PLGA–PEI–PEG–arginylglycylaspartic acid at higher N/P ratios, but opposite is ture at low N/P ratios (181). In a study, the combination of poly L-lysine (PLL) with PLGA in PLGA/PLL nanoparticles demonstrates an increased stability of the Ebola DNA vaccine delivered using a microneedle patch, without inducing cytotoxicity as both materials are biocompatible (182). PLL/PLGA increases the water solubility of nanoparticles and even modifies the surface charge to promote DNA encapsulation and transfection efficiency (183). Additionally, mPEG-PLGA-PLL nanoparticles successfully deliver epidermal growth factor (EGF) and Bcl-2-siRNA genes to target H1299 lung cancer cells, resulting in inhibited tumor growth by reducing Bcl-2 expression in tumor tissues. The nanoparticles exhibit efficient transfection with low cytotoxicity, as demonstrated by the MTT assay. To address the issues of insufficient charge and low transfection efficiency in gene delivery using single PLGA nanoparticles, chitosan/PLGA (modified with chitosan) has been developed, which improves the overall performance (184).

In summary, PLGA is a safe and effective carrier for biomedicine, particularly in nucleic acid delivery to target cells, owing to its biocompatibility and biodegradability. Although there are challenges such as low encapsulation efficiency and slow DNA release, advancements in DNA nano-encapsulation technologies and the incorporation of various organic or inorganic components are being explored to overcome these limitations. The significant progress made in the improvement of PLGA-based nano-delivery systems has led to the successful treatment of animal disease models. *In vivo* and *in vitro* studies have consistently demonstrated the potential advantages of these systems, highlighting the importance of rational design in the field of biomedical engineering.

### 3.5 Exosomes

Exosomes are naturally occurring nanoscale particles secreted by both eukaryotic and prokaryotic cells. They typically range in size from 30 to 150 nanometers. Comprising cellular derivatives such as proteins, lipids, genetic information, cytokines, and growth factors, exosomes play crucial roles in immune regulation, intercellular communication, and inflammatory responses (185). Furthermore, exosomes are widely present in various bodily fluids, such as blood, urine, cerebrospinal fluid, among others and transport a diverse array of vital biological signaling molecules, including recombinant proteins, RNA, and DNA (186).

Exosomes, as natural nanocarriers for DNA delivery, offer several advantages. These advantages include low immunogenicity, extended circulation time in the bloodstream, and the ability to traverse the blood-brain barrier. In a notable study by Kim et al., exosomes derived from ovarian cancer were successfully employed as carriers for CRISPR/Cas9 DNA to effectively suppress the expression of poly (ADP-ribose) polymerase 1(PARP-1) in a xenograft mouse model (187). As a result, exosomes hold significant promise as potential vehicles for DNA-based therapeutics. Their inherent stability, biocompatibility, and capacity to cross biological barriers, including the blood-brain barrier and gastrointestinal barrier, make exosomes well-suited for reshaping DNA drug delivery. Exosomes are often regarded as “nature’s lipid nanoparticles, which possess specific ligands or adhesion molecules on their membranes that facilitate DNA release through interactions with recipient cell membranes or intracellular uptake (188). Consequently, exosomes can serve as effective nanocarriers for nucleic acid vaccines. In comparison to liposomes or other polymer-based nanoparticles, exosomes contain transmembrane and membrane-anchored proteins that enhance endocytosis, facilitating efficient vaccine delivery (189). Additionally, immune cells, such as monocytes and macrophages secrete exosomes that can evade immune phagocytosis, further enhancing their potential as carriers for DNA-based therapeutics and vaccines (190).

The lipids, proteins, and nucleic acid-like signaling molecules present on the surface of exosomes also actively participate in T-cell activation and immune modulation. Several studies have reported that exosomes containing DNA, released by E0771 cells and delivered to GM-DCs’ cytoplasm, can activate the STING-dependent pathway, thus optimizing anti-tumor immunity (191). This highlights the growing significance of cell-derived exosomes as a novel platform for vaccine delivery, which is in high demand in vaccine research. Recent research has underscored the critical role of exosome-based vaccines in eliciting immune responses. These responses encompass various aspects, including antigen presentation, the induction of humoral immunity, activation of cellular immunity, cytokine secretion, and antigen clearance (189). Notably, exosome-based vaccines tend to favor a T-helper cell type 1 (Th1) immune response, which provides superior protective immunity (192). Exosome vaccines carrying tumor DNA antigens have the potential to induce anti-tumor immune responses by delivering these antigens to antigen-presenting cells (APCs) like dendritic cells (DCs), potentially obviating the need to purify tumor antigens. Studies have suggested that genetically engineered exosomes derived from tumor cells expressing early secreted antigenic target-6 (ESAT-6) could serve as promising candidates for cancer vaccines (193). This strategy has the potential to significantly enhance host defense by restoring cytotoxic T lymphocyte (CTL) responses and promoting viral clearance, addressing the challenge of chronic infectious diseases. In recent developments, a novel exosome-based adjuvant delivery system was created using genetically modified mouse melanoma B16BL6 cells. In this system, exosomes from these cells contained CpG DNA and successfully induced immune-stimulating signals in mice after the final immunization (194). This breakthrough opens up new avenues for using exosomes as carriers for adjuvants in future cancer vaccine development.

However, it’s important to acknowledge that research on exosome-based drug delivery encounters several challenges. Firstly, achieving large-scale production of exosomes for clinical trials remains a major hurdle. A cellular nanopore method developed by Yang et al. has shown promise in producing high-yield exosomes, surpassing traditional methods by 50 times (195). Secondly, the development of efficient methods for loading DNA vaccine into exosomes is crucial. Conventional loading strategies often suffer from low efficiency, which can potentially lead to nucleic acid aggregation, degradation, and alterations in exosome properties (196). The complex tumor microenvironment and the heterogeneity of exosomes may also impact exosome delivery efficiency. Therefore, further research is needed to fully elucidate the exact biological functions of exosomes. As we move forward in the realm of vaccine development, new platforms such as DNA, mRNA, and active carrier technologies have emerged, overcoming some limitations of traditional vaccines and allowing for faster production. Each vaccine platform has its unique advantages and disadvantages, and it is unlikely that a single technology will suffice to protect humans from all cancer and infectious disease threats. Therefore, there is a need to rethink how to make currently available vaccines more effective, with one emerging targeted vaccine delivery platform involving exosomes. Understanding the role of exosomes in immune responses and pathological mechanisms for many lethal diseases will be a crucial challenge in harnessing exosomes for vaccine development.

In conclusion, research into exosome-based drug delivery holds immense promise while also presenting formidable challenges, including the need for scaling up production, improving drug loading efficiency, and navigating the intricacies of the tumor microenvironment. Nonetheless, as an emerging field, exosome-based drug delivery opens doors to exciting prospects for advancing the field of medicine.

### 3.6 VLPs and microalgae

Virus-like particles (VLPs) are non-infectious structures that mimic the organization and conformation of native viruses but lack the viral genetic material (197), making them safe for use in vaccines. When it comes to delivering DNA vaccines, VLPs can serve as an effective and versatile delivery platform (198). The VLP serves as a carrier for the DNA, and protect it from degradation and facilitating its delivery to target cells (199). VLPs with the enclosed DNA vaccine can enter cells through endocytosis and/or other mechanisms (200). Using VLPs as delivery vehicles for DNA vaccines offers several advantages, including enhanced stability of the genetic material, improved cellular uptake, and the ability to stimulate both humoral and cellular immune responses (198). Additionally, VLPs can be designed to mimic the structure of specific viruses, eliciting a more targeted and effective immune responses (201).

Moreover, the application of microalgae in delivering DNA vaccines represents an emerging and promising frontier in biotechnology. Microalgae, like *Chlamydomonas reinhardtii* (202), exhibit several advantages that position them as attractive candidates for DNA vaccine delivery. First, microalgae are generally recognized as safe (GRAS) (203), posing no risk of infection to humans, making them a secure choice for vaccine production. Second, microalgae can be cultivated easily in large-scale bioreactors, providing a cost-effective platform for vaccine manufacturing (204). Third, microalgae can undergo post-translational modifications, including glycosylation, similar to higher eukaryotes. This is crucial for the proper folding and functionality of certain proteins, including those used in vaccines (205). Fourth, stability and shelf life are notable benefits, as algal cells can be stored for extended periods without losing viability, ensuring a prolonged shelf life for the vaccines. Fifth, as photosynthetic organisms, microalgae utilize light as an energy source for protein production, contributing to an environmentally friendly and sustainable approach to vaccine production (206). Lastly, the ease of oral administration is a significant advantage for DNA vaccines, eliminating the need for injections and facilitating broader vaccine distribution (207). The typical process involves introducing the necessary DNA sequence encoding the vaccine antigen into microalgal cells. Like other strategies, subsequently, these cells express and produce the antigen upon administration, and can trigger an immune response. Researchers are actively working to optimize various aspects of this technology, such as increasing expression levels, ensuring proper protein folding, and enhancing the efficiency of oral administration.

While the use of VLPs or microalgae for DNA vaccine delivery holds promise, the research is still in infancy, and practical applications needs to be studied more.

### 3.7 Electroporation

In the delivery of DNA plasmid, the primary objective is to achieve a high concentration of DNA within the nucleus. However, the cell surface membrane, cytoplasm, and nuclear envelope pose limitations to the efficient uptake and transport of naked DNA into the cells. Electroporation is a technique that utilizes brief electric impulses to temporary disrupt the cell membrane, facilitating the efficient entry of macromolecules, including DNA, into cells both *in vitro* or *in vivo* (208). This technique is highly versatile and can be optimized for different nanoparticle-sized carriers by adjusting parameters such as voltage, number of electrodes, depth, cell types, DNA, and animal models. Electroporation can significantly enhance the transgene expression by 10 to 1000-fold, and has the potential to overcome the delivery barrier of transgene in large animals (209, 210). In addition to its role in facilitating gene delivery, electric pulse alone are known to trigger robust immune responses to solid tumors. Electroporation induces minor inflammation at the injection site without significant adverse effects (211, 212). It does so by inducing an adjuvant-like response, recruiting T-lymphocytes, and upregulating cytokines, as evidenced by increased levels of pro-inflammatory cytokines (IL-1β and TNF-α) (213). In cancer therapies, electroporation involves the study of tumor ablation using irreversible electroporation (214), CAR-T transfection (215), gene therapies (216), and vaccinations (217). Consequently, it has garnered significant attention from cancer immunologists seeking to improve their treatment strategies.

Electroporation can be reversible if the electric pulses remain within the adaptive capacity of the specific cells. Clinical trials have demonstrated that electroporation not only enhances the cellular immune response but also extends the duration and the range of response to multiple antigens (218). This technique enables the introduction of naked DNA plasmids into muscular cells. Naked DNA molecules are typically susceptible to degradation by nuclease and have difficulty-entering cells due to their hydrophilic properties. However, when the electric pulses exceed the threshold of approximately 3 kV/cm for 1-100 milliseconds, they can disrupt the nanoscale membrane, leading to cell apoptosis (219). The membrane permeability and intracellular effects can be modulated by adjusting parameters such as voltage, electric pulse frequency, and electric pulse duration. The specific pulse duration and applied field strength may vary depending on the intended application. In traditional gene delivery, longer pulses in the millisecond range are commonly used at lower voltages (approximately 8 kV/cm) for DNA transfection *in vitro* (220).

Indeed, the use of nanosecond pulses in electroporation is gaining attention for studying intracellular delivery of DNA, particularly for facilitating the entry of DNA into the nuclear membrane in addition to the cell membrane. Advanced pulse generators capable of producing hundreds of kilovolts per centimeter (kV/cm) pulses have allowed researchers to investigate the effects of electroporation on intracellular processes, such as calcium release, apoptosis, nuclear morphology, and stress response. Napotnik and her research group demonstrated that optimal electroporation of the plasma membrane requires longer pulse durations, while intracellular electroporation is more efficient with shorter pulse durations, taking into account the electrophoretic aspect of DNA migration (221). The concept is further supported by the observation that increasing the electric pulse frequency enhances transfection efficiency from 1 Hz to 1 MHz (200 ns pulses at 10-18 kV/cm). However, higher electric pulse frequencies also lead to decreased cell viability (222). Therefore, a combination of millisecond and nanoseconds electroporation may offer a means to achieve high-throughput DNA delivery through both the plasma membrane and nuclear membrane. Recently, researchers have incorporated microfluidic into the electroporatation process to mitigate issues, such as Joule heating and gas bubbling associated with nanosecond pulse treatments. By combining nanosecond pulses with millisecond pulses in a staggered manner, they achieve higher transfection efficiency while maintaining cell viability (223). It should be noted, however, that the impact of electroporation on chromosomes and disruption of basic organelles, aside from gene delivery, still require further elucidation. Ideally, a favorable electric field range can be identified that enhances gene migration through the nuclear envelope without a huge impact on cellular homeostasis.

As electroporation is a physical penetration method, it can be used alongside carriers to further enhance transfection efficiency. However, the electric pulse can disrupt the cellular membrane, it may simultaneously decrease the encapsulation efficiency of the carriers. Nevertheless, a study conducted in pigs demonstrates that electroporating DNA-encapsulated cationic nanoparticles made of PLGA results in greater inflammation and immunity compared to electroporating naked plasmid and using microneedle patches (224). This approach shows good transgene expression and elicits both cell-mediated and humoral immune responses.

### 3.8 Other physical delivery systems

With the increasing demand for gene delivery in various fields, such as genetic studies, gene editing, transfection, and vaccinations, more methods have been explored to enhance the outcome. Gene guns employ the biolistic method for transfection, in which carriers coated with nucleic acids are propelled at high velocity towards cells. Typically, gold or tungsten carriers are used due to the inert properties. The shape of the carrier also plays a significant role as it affects the transdermal penetration ability, with gold nanorods demonstrating superior performance compared to gold nanospheres. The three crucial components of gene gun delivery include: DNA-coated carriers, insertion of carriers into cartridges, and discharge of carriers using high gas pressure (225). The needleless gene gun method can be used both *in vivo* and *in vitro*. In a study on HPV DNA vaccines, gene gun delivery resulted in approximately a 4-fold increase in CD8^+^ T cell precursor compared to intramuscular injection, although its immunogenicity is still incomparable to the electroporation (226). However, researchers also observed higher levels of circulation with gene guns than with other methods.

Needleless injection reduces waste and minimizes the risk of accidental injuries. Another needle-free method is the jet injector, which employs high-pressure fluid to penetrate the skin. It offers advantages over traditional needle injection, especially in mass immunizations. Multi-use-nozzle jet injectors (MUNJIs) initially gain popularity due to their reusability, enabling repeated injections with the same nozzle (227). Consequently, they expedite the vaccination process, reduce costs, and minimize discomfort. However, concerns arose regarding cross-contamination or cross -infections associated with reusing vaccination devices. To address this, disposable-cartridge jet injectors (DCJIs) were introduced, which eliminates the risk of using a new drug cartridge for each injection. The new design also addresses the issue of blood backsplash onto the nozzle. In DNA vaccinations, malaria antibody titers in laboratory rabbits indicate a 10 to 50-fold increase with jet injection compared to needle injection (228). As SARS-COV-2 DNA vaccine injection with electroporation enters clinical trials, DCJIs are being considered as an alternative to electroporation due to the logistical benefit of disposable cartridges (229). Researchers have also attempted to combine jet injection and electroporation into a single DNA vaccine treatment, as electroporation can serve as a physical adjuvant. Multiple instances of jet injection combined with electroporation have been shown to significantly induce both humoral and cellular immunity (230). As justified that electroporation can increase DNA uptake by cells through membrane disruption, the jet injection can also achieve that by improving the dispersion of vaccine over a larger tissue area with high pressure (231). These complementary improvements propose a better DNA delivery method for targeting cells.

Microneedle patches deliver plasmid DNA primarily through the intracutaneous pathway. These biocompatible devices consist of numerous micron-sized dips that are coated with a drug capable of slow release across the stratum corneum. This method requires a high concentration of pDNA on the surface of the microneedle and is minimally invasive compared to traditional needle injections. Furthermore, it allows for sustained co-delivery of multiple drugs, eliminating the need for multiple doses (232, 233). Microneedles can be coated with the drug or contains the drug if the material is biodegradable. As immunization alone does not facilitate the migration of DNA vaccines through the cell membrane, it requires nanocarriers, such as PEI or PLGA to facilitate the transportation (234). The length of the microneedles can be manipulated to allow targeted drug delivery to specific skin layers, reducing errors and preventing damage to nerves and vascular structures (235). In summary, dermal administration through electroporation provides a significant number of APCs, resulting in enhanced immune responses, particularly when immune cells are the target, such as in the case of checkpoint molecules (e.g. PD1, CD80/86). Moreover, it serves as a safe, painless, and self-administered delivery system that can enhance the overall immune response, against the desired target in the organism, thereby augmenting population immunity itself.

## 4 Expert opinion

Gene therapy, particularly DNA vaccine, is poised to play an increasingly significant role in future medicine due to its stability, cost-effectiveness, high-yield, safe administration, and simple manufacturing. With an effective delivery system, the carrier can serve as an efficient Trojan Horse, delivering beneficial DNA into host’s cells, transmitting information, and activating immune responses. However, achieving the optimal delivery system requires a comprehensive consideration of cost, cell selectivity, toxicity, vaccine-induced immunogenicity, and off-target effects. DNA vaccines for cancer treatment and prevention are particularly promising. Although several DNA-based vaccines have been approved by the FDA and USDA for preventing and treating veterinary diseases, there have been no FDA-approved DNA vaccines for human diseases. The current level of response is insufficient to effectively resist and cure diseases and provide clinical benefits. Therefore, optimizing DNA vaccine strategies and DNA delivery systems pose significant challenges in improving their efficacy and effective DNA delivery into the nucleus for expression and uptake by APCs is crucial for eliciting an effective immune response. Over time, viral vectors such as adenoviruses and retroviruses, as well as non-viral vectors like liposomes, PEI, chitosan, and PLGA, have been developed for the development and application of DNA vaccines. However, viral vectors carry the risk of gene integration, induce highly immunogenic off-target effects, and may have cellular and tissue toxicities. Non-viral vectors, on the other hand, exhibit unsatisfactory gene delivery efficiency, limited capacity for DNA loading, low biocompatibility, and susceptibility to DNA damage and degradation, which limit their clinical application in DNA vaccines. Nevertheless, advancements in nanotechnology and biomedical applications have made it possible to personalize and optimize the design of DNA vaccine delivery systems.

NPs synthesized from various materials offer opportunities for tailoring the composition, size, shape, properties, and surface modification of particles. As DNA delivery vehicles for DNA vaccines, NPs can protect DNA from degradation, accommodate large DNA fragments, enhance biocompatibility, utilize degradable materials to reduce carrier toxicity and enable targeted delivery to specific cells or tissues (e.g., APCs) through ligand-receptor modifications. Nano-vaccines are a newer type of vaccine that utilizes nanomaterials to deliver plasmids or antigens to the immune system. Compared to traditional DNA vaccines, which introduce naked genetic material into cells to produce antigens, nano-vaccines have several advantages in terms of their lymph node accumulation, antigen assembly, and antigen presentation. One key advantage of nano-vaccines is their ability to accumulate in lymph nodes, which are crucial sites of immune response. Because nanomaterials are often small enough to be taken up by APCs, they can be efficiently transported to lymph nodes where they can activate immune responses. This can lead to stronger and longer-lasting immune responses compared to traditional DNA vaccines. Another advantage of nano-vaccines is their ability to assemble antigens into specific structures that can optimize immune responses. For example, some nano-vaccines use self-assembling peptides or proteins to form precise structures that mimic viral particles. This can improve the ability of the immune system to recognize and respond to the antigen, leading to more effective protection against infection. In addition, nano-vaccines can also enhance antigen presentation to immune cells. By using nanomaterials to deliver antigens directly to DCs, the vaccine can more efficiently activate the immune system and generate a stronger response. This is particularly important for vaccines targeting pathogens that are difficult to neutralize, such as viruses with high mutation rates. While DNA itself acts as vaccine adjuvant (through cGAS-STING-type I IFN pathway), NPs can also incorporate auxiliary drugs or adjuvants to enhance immunogenicity and induce specific immune responses. They achieve controlled release characteristics of DNA for long-lasting immune responses, and facilitate nuclear delivery through the modification of nuclear localization signal. Despite the evident advantages of NP delivery systems, further optimization is required to address issues related to NP distribution, retention by the endothelial reticulum system, DNA release, and efficient delivery across nuclear membranes. Nevertheless, NP-based delivery has emerged as a prominent area of research. Moreover, the development of integrated DNA vaccine delivery systems that combine the complementary advantages of traditional delivery vehicles is becoming a growing trend.

When comparing DNA vaccines to recombinant protein vaccines, aside from the manufacture and storage of the biological drug, the former can induce strong cellular immune response, but they are generally less effective in inducing a robust humoral immune response compared to recombinant protein vaccines. Therefore, a current hotspot in the field involves the development of the synergistic immunity using a combination of DNA and recombinant protein vaccines to overcome these limitations. One concern regarding DNA vaccines is the magnitude and duration of the humoral immune response. The factors influencing the magnitude and durability of the immune response include the specific antigen used, the delivery method, the formulation of the vaccine, and the individual’s immune system characteristics. Immune durability, or the ability of the immune response to provide long-lasting protection, is another important aspect of vaccine efficacy. DNA vaccines have shown varying degrees of immune durability in different studies and for different diseases. Some DNA vaccines have demonstrated long-lasting immune responses, while others have shown a need for booster doses to maintain protective immunity. To address these concerns and improve the humoral immune response and immune durability of DNA vaccines, researchers are exploring various strategies. These include optimizing the delivery methods, using adjuvants to enhance the immune response, and developing novel DNA vaccine formulations. It is important to note that the field of DNA vaccines is evolving rapidly, and ongoing research and clinical trials are continuously expanding our understanding of their efficacy, safety, and immune responses. Future advancements may help overcome the current limitations and improve the humoral immune response and immune durability of DNA vaccines.

Another concern regarding DNA vaccines is the possibility of complete or partial genome integration. Nevertheless, the existing data indicates that the integration rate is exceedingly low, even less than the rate of spontaneous mutagenesis. The DNA proofreading mechanism is ahead of to any other mechanism for ensuring the integrity of the host genome (236). Although, not proved scientifically, induction of antibiotic resistance in the host upon DNA vaccination. This belief is due to the insertion of antibiotic resistance gene into the plasmid construct. To avoid misconception, the researchers have come up with the novel DNA construct called “doggybone” DNA, which is synthesized by the DNA amplification system free from bacterial cells (237). The efficacy of DNA vaccines is significantly influenced by the choice of delivery system and route of administration. Different delivery systems, including viral vectors, liposomes, or other nanotechnologies, are influenced by vaccine characteristics, target populations, and application scenarios (238).To optimize the efficacy of DNA vaccines (or any other class of vaccines), thorough exploration and research into various delivery systems, as a perfect mix, are imperative. This strategic approach enables the development of flexible and efficient vaccination strategies, tailored to diverse application scenarios.

Moreover, various administration routes of DNA vaccines, such as intramuscular, subcutaneous, or mucosal, may affect the type and magnitude of immune responses (239). Different DNA vaccine administration routes exhibit variations in eliciting immune response (239). Compared to intramuscular and subcutaneous injections alone, the combination of the two results in an increased antigen-specific CD4^+^ and CD8^+^ T cell response (240). Optimizing the administration route involves careful consideration of vaccine characteristics and desired outcomes. Choosing the most appropriate route for specific vaccines and effects, along with exploring novel administration approaches, represents a strategic avenue for enhancing the overall efficacy of DNA vaccines. The synergy between the delivery system and administration route is pivotal in determining the success of DNA vaccines. Thorough understanding, strategic selection, and continuous optimization of these factors are crucial for advancing the field and realizing the full potential of DNA vaccines in diverse medical applications.

It is vital to recognize the limitations and distinctions across species, particularly in establishing and breaking tolerance. While demonstrating these concepts in mice may be more straightforward, translating such findings to higher species, including humans, demands meticulous consideration. The wealth of experience gained from oral DNA vaccine studies in preclinical animal experiments can serve as a valuable guide to address the current bottlenecks in clinical applications. As technology advances and updates are made, the need for additional research and validation in higher species will become even more apparent. The ultimate goal is to develop effective oral DNA vaccines for a broad spectrum of recipients, including livestock, pets, and humans, by overcoming various challenges. Doing so can ensure a more accurate and reliable application of the concepts discussed in the study of oral DNA vaccines. This ongoing research and development are essential to realizing the full potential of oral DNA vaccines and bringing them into practical use for the benefit of diverse populations. Regarding several times more doses required for oral vaccines in countries with enteric diseases like Bangladesh compared to Europe or the USA, several factors may contribute to this difference, including environmental conditions, immune history, nutritional status, pathogen diversity, and healthcare infrastructure. Researchers and public health officials need to consider these factors when designing vaccination strategies and dosing regimens tailored to specific regions and populations. Additionally, ongoing monitoring and adaptation of vaccination programs based on real-world effectiveness are crucial for ensuring optimal vaccine coverage and protection.

Aluminum salts, commonly referred to as alum, have been widely used as adjuvants in vaccines to enhance the body’s immune response to the antigen for many decades and are considered the gold standard of adjuvants (241). They can induce macrophages to transform into APCs (242). Additionally, a short-term reservoir is formed at the injection site, slowly releasing antigens and prone to phagocytosis, thereby enhancing the immune mechanism (243). LNP, serving as a carrier for DNA vaccines, can express antigens and activate the immune system (243), but it does not have the function of an adjuvant. However, both alum and LNP play crucial roles in immunological vaccines, yet their functions and application areas differ. In the design of vaccines, the appropriate adjuvant may need to be selected based on factors such as the target, immune mechanism, and safety. In some cases, combining different adjuvants might also be possible to achieve a more potent immune effect.

## 5 Conclusion

In summary, DNA vaccines have emerged as an important tool in the prevention and treatment of modern biomedical diseases. However, the development of an efficient delivery system remains the most critical challenge in DNA vaccine research. The potential of DNA vaccines to revolutionize the treatment of human diseases is vast. One significant issue in the field is the lack of standardized methods for comparing DNA vaccination approaches. Factors such as dosage, encapsulation conditions, and types of DNA structures vary across studies. To ensure progress in the field, it is crucial for researchers to strive for reproducibility in DNA delivery methods. Efficient encapsulation of nanoparticles using conventional techniques such as microfluidics, double emulsification, and multi-inlet vortex mixing can lead to faster and more effective production of DNA-based biological drugs. Furthermore, the choice of delivery system and administration route can significantly impact the efficacy of DNA vaccines. Experimentation and further research are needed to determine the optimal injection site and the use of injection devices. Collectively, the development or optimization of effective delivery systems for DNA vaccines has the potential to unlock new frontiers in immunotherapy.

## Author contributions

BL: Writing – original draft, Writing – review & editing. JL: Writing – original draft, Writing – review & editing. BY: Data curation, Writing – review & editing. SS: Investigation, Writing – review & editing. PN: Writing – review & editing. NS: Writing – review & editing. PK: Writing – review & editing. SB: Writing – review & editing. RK: Writing – review & editing. JZ: Supervision, Writing – review & editing. DC: Conceptualization, Funding acquisition, Supervision, Writing – review & editing.
